# Comparison of mask R-CNN and YOLOv8-seg for improved monitoring of the PCB surface during laser cleaning

**DOI:** 10.1038/s41598-025-02131-7

**Published:** 2025-05-17

**Authors:** Lu Qiao, Markus Veltrup, Bernd Mayer

**Affiliations:** 1 Department of Plasma Technology and Surfaces, Fraunhofer Institute for Manufacturing Technology and Advanced Materials, 28359 Bremen, Germany; 2 Faculty for Production Engineering, University of Bremen, 28359 Bremen, Germany

**Keywords:** Machine vision, Deep learning, PCB recycling, Laser cleaning, YOLOv8-seg algorithm, Mask R-CNN algorithm, Engineering, Materials science, Mathematics and computing

## Abstract

Potting compounds and coatings protect electronic components in harsh environments, requiring careful removal for recycling or repair. This study introduces the innovative use of YOLOv8-seg and Mask R-CNN to enhance the precision and efficiency of the laser cleaning process for PCBs (Printed Circuit Boards). These models are utilized for two primary tasks: real-time segmentation for laser cleaning guidance and post-cleaning surface quality assessment. Real-time segmentation adapts cleaning strategies based on PCB surface states such as ‘Bare-Cu’, ‘Complete-Removal’, ‘Incomplete-Removal’, etc. Quality assessment ensures high-quality, damage-free surfaces post-cleaning. Both models were trained on an augmented dataset to improve robustness. In the initial test dataset, YOLOv8-seg (l), known for its speed, achieved an mAP50 (seg) of 82.8% at 3.98 FPS, proving suitable for time-sensitive laser cleaning processes due to its speed and precision. Mask R-CNN (ResNet-50) reached an mAP50 (seg) of 84.097% at 1.52 FPS, fulfilling real-time requirements with high precision. Although their visualization segmentation results on the initial test dataset vary, both models successfully address the previously mentioned tasks. When tested on a new dataset with unseen patterns it was shown that YOLOv8-seg excels at generalizing to new patterns while Mask R-CNN performs less effectively. This study confirms YOLOv8-seg’s effectiveness in real-time PCB monitoring during laser cleaning, boosting automation and efficiency in PCB recycling.

## Introduction

The “European Green Economy Deal” (2019) and the “Circular Economy Action Plan” (2020) emphasize sustainability in sectors such as battery technology and electronics^1^. As part of these initiatives, the EU mandates an electronic product passport and a “Right to Repair” which requires products to be durable and repairable^2^. Protective coatings made from special potting resins are designed to protect components from environmental factors such as moisture, chemicals, and temperature fluctuations. However, these coatings prevent the effective repair and recycling of PCBs. Compared to traditional mechanical and chemical cleaning techniques^3^, laser cleaning provides a versatile solution due to its wide application range, enhanced environmental safety, and significantly reduces the generation of hazardous substances^4–6^. However, the efficiency of laser cleaning can be compromised by the uneven thickness of coatings, diverse surface geometries, and the complex interactions between thermal and mechanical factors which sometimes lead to substrate damage or incomplete cleaning^6^. These issues highlight the need for advanced monitoring technologies to ensure high-quality laser cleaning processes.

Traditional laser cleaning monitoring primarily relied on human judgement for defect detection, making it resource-intensive and inefficient^7^. There has been a shift towards employing various sensors to monitor the laser processing, such as acoustic monitoring^4,8^, optical monitoring^9–12^, and machine vision systems based on image processing methods^13^. Among these methods, integrating deep learning (DL) algorithms with vision systems stands out for its superior adaptability and precision. This is due to their ability to learn from large datasets, recognize complex patterns, and continuously improve through training. As a result, researchers have increasingly focused on these integrated approaches, leading to more effective and precise methods for defect detection and surface analysis in the intelligent manufacturing industry. Chen et al.^14^ developed a YOLOv4-based model to identify steel plate stains before laser cleaning. Both Junlong et al.^15^ and Hu et al.^16^ made significant advancements in PCB defect detection, with Junlong et al. enhancing the YOLOv5 algorithm and Hu et al. integrating Faster R-CNN with a Feature Pyramid Network. Furthermore, Zhu et al. by using a CMOS-Sensor^17^ and Kriegler et al. using a laser confocal microscope^18^ applied advanced segmentation algorithms for defect detection and quality evaluation in laser welding and laser cutting processes within battery production, respectively. Wu et al.^19^ also utilized Mask R-CNN for precise solder joint recognition.

The studies above highlight the crucial role of DL algorithms in the manufacturing sector. The objective of this study is to address a notable research gap by advancing beyond the current state of art. In contrast to conventional approaches which are limited to defect detection on uncoated PCBs, this paper presents a real-time monitoring of the laser ablation of the protective layer on PCBs. This is achieved without damaging the underlying PCB, thereby enabling the automatic repair of coated boards for the first time. A significant challenge is the efficient processing of data in a time frame of less than 700 ms which is essential in industrial applications to enable prompt process adjustments. Furthermore, the detection of PCB defects in industrial settings presents significant challenges, including inadequate lighting, dust, and surface contaminants, which can obscure features and negatively impact segmentation accuracy. Additionally, the seamless transition between the removed protective layer and the remaining one, which occurs during decoating, can be challenging. Moreover, the PCB comprises numerous potential classes that must be identified during or after laser decoating. To detect the coating residues on the various components of the PCB and evaluate the progress of the removal, we compared the algorithms for segmenting instances algorithm YOLOv8-seg^20^ and the Mask R-CNN^21^ with regard to their performance in terms of accuracy and computing time. Alternative instance segmentation algorithms, such as DeepLab^22^, PANet^23^, and SOLO^24^, were considered but not chosen due to limitations such as a lack of native instance segmentation capabilities, higher computational complexity, or reduced practicality for real-time PCB monitoring. The YOLOv8-seg model, derived from YOLOv8. YOLOv8 achieves strong accuracy on COCO (e.g., the YOLOv8m model achieves a 50.2% mAP when measured on COCO^25^), retains the rapid processing characteristics of one-stage models, rendering it suitable for future real-time applications. The Mask R-CNN model, built upon Faster R-CNN, delivers outstanding performance on the COCO test set. Leveraging ResNet-101 as its backbone, it achieves a mask average precision of 35.7% at a processing speed of 5 FPS, outperforming all previous state-of-the-art single-model results, for instance, segmentation on COCO^21^. By incorporating pixel-level segmentation, it becomes highly effective for applications demanding precise object localization and segmentation. This study aimed to validate whether YOLOv8-seg, renowned for its real-time processing capabilities, could meet the precision requirements for segmenting the surface states of PCBs. Similarly, we assessed if the Mask R-CNN, known for its high precision in segmentation tasks, could deliver the necessary processing speed to fulfil future real-time requirements. Their performance was compared based on several criteria, including loss metrics, mAP50, processing speed, and the segmentation visualization results for the initial and new test datasets. By analyzing these factors, we identified the most suitable DL model for monitoring the laser cleaning process, balancing precision and processing speed requirements to ensure efficient and reliable monitoring of PCB surface states.

The selected DL algorithm will be used to develop a real-time monitoring system for PCB laser cleaning. High-resolution images from the image acquisition unit feed into DL algorithms, enabling accurate identification of coating residuals. This real-time analysis will allow immediate adjustments to laser cleaning parameters, ensuring optimal performance and preventing substrate damage. Furthermore, by segmenting the PCB surface area, the DL algorithm can assess the quality of the laser cleaning. The synergy between DL and vision systems results in precise defect detection, dynamic process adjustments, and continuous optimization, significantly improving the efficiency and effectiveness of the PCB laser cleaning process.

The structure of this study is as follows: 1. Materials and Methods provides an overview of the laser cleaning system, image acquisition, dataset preparation, training devices, and model evaluation metrics. 2. Results and Evaluation presents the performance results of the model metrics and visualizes the segmentation results for both the initial and new test datasets. 3. Discussion evaluates the best-suited model, discusses study limitations, and suggests directions for future research. 4. Conclusion summarizes the key outcomes and highlights potential advancements in laser cleaning monitoring systems.

## Materials and methods

### Laser cleaning system

**Figure 1:**
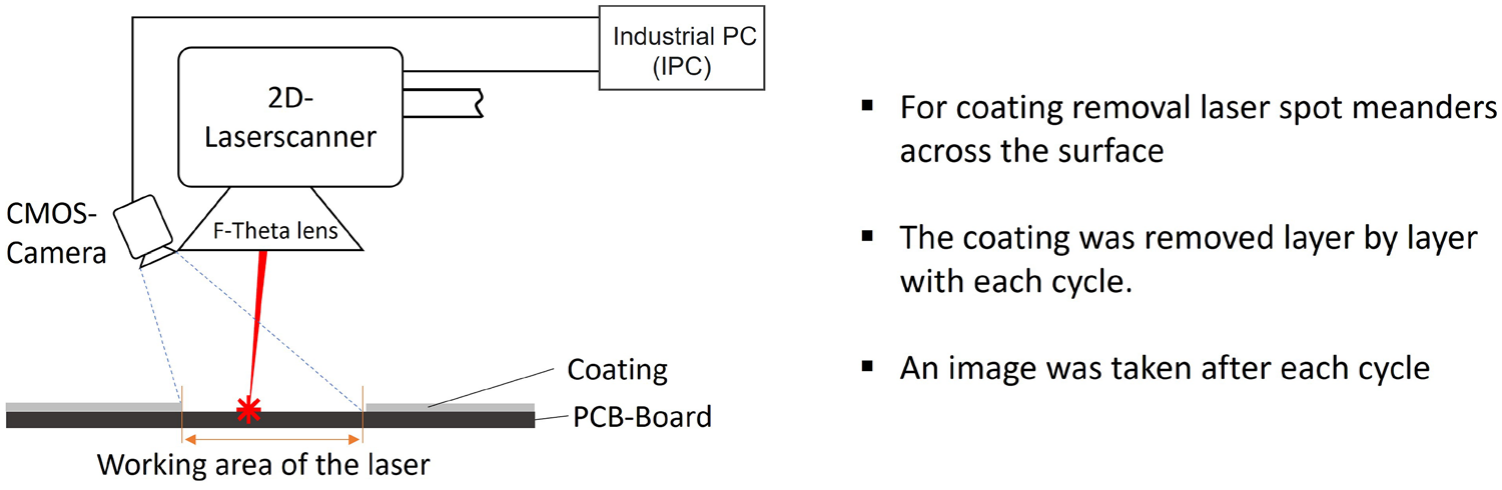
Schematic test setup for the laser-based coating removal.

Treatments were performed using an infrared fibre laser with a wavelength of $\lambda$ = 1064 nm of the type CL 20 from Clean-Lasersysteme GmbH (Herzogenrath, Germany) with a spot diameter of 100 $\mu$m full width at half maximum (FWHM) by using an F-Theta lens (f = 330 mm). The pulse duration of the laser was approx. 105 ns. The systems generate pulse frequencies in the range of 40 kHz to 200 kHz. For the presented experiments, the laser spot meanders across the surface. Line spacing and pulse overlap were calculated based on the spot diameter and pulse frequency, and a laser power of 16 W at 40 kHz was used. The line spacing was 20 $\%$ and pulse overlap 95 $\%$. The decoating process entailed the removal of the protective layers in successive cycles, with a time interval of approximately 700 ms between each cycle. To measure the time interval, the time scales for readjusting the process were determined experimentally and correspond to the duration required by the 2D laser scanner to initiate the subsequent cycle after completing a full scan cycle. This time includes the repositioning of the two galvo scanners to the starting point of the processing field and the initiation of the subsequent laser processing cycle (triggering of the laser). Control signals were monitored and analyzed to determine these time intervals. This interval was utilised to capture and process an image of the treated surface. The treatment was cancelled if it turned out that the component had been overtreated. However, a maximum of 25 cycles were carried out. Figure 1 provides a schematic representation of the test setup.

### Image acquisition

A Basler Ace 2 camera^26^ was used in the image acquisition unit to gather 700 PCB surface images after each laser cleaning operation cycle was completed. The camera features a Sony IMX392 CMOS sensor with a global shutter. It offers a resolution of 2.3 MP (1920 x 1200 px) and a framerate of 51 FPS for precise color imaging in the visible spectrum. We optimized the light sources by using professional photography fill lights and reflectors, positioning them to provide even illumination across the PCB surface and significantly reduce shadows and under-illumination.

### Images annotation, preparation for model training

#### Images annotation

To facilitate real-time monitoring and quality assessment during the laser cleaning of PCBs, the states of the PCB surface are categorized into five specific areas: Bare-Cu, Complete-Removal, Incomplete-Removal, Partial-Coating-Cu, and Severe-Damage. These labels and their corresponding PCB surface states are as follows:

- Bare-Cu: Bare-Cu is distinguished by completely removing the coating from copper traces and exposing the copper surface.
- Complete-Removal: In contrast to Severe-Damage, the Complete-Removal category is characterized by minor PCB substrate damage following the complete removal of surface coatings.
- Incomplete-Removal: This category is defined as incompletely removing the surface coating on the PCB substrate after laser cleaning.
- Partial-Coating-Cu: Partial-Coating-Cu signifies instances where the cleaning process leaves behind a fraction of the coating on the copper surface, thus not fully exposing the copper surface.
- Severe-Damage: This category indicates PCBs where the substrate exhibits extensive overtreatment after completely removing surface coatings via laser cleaning.

**Figure 2:**
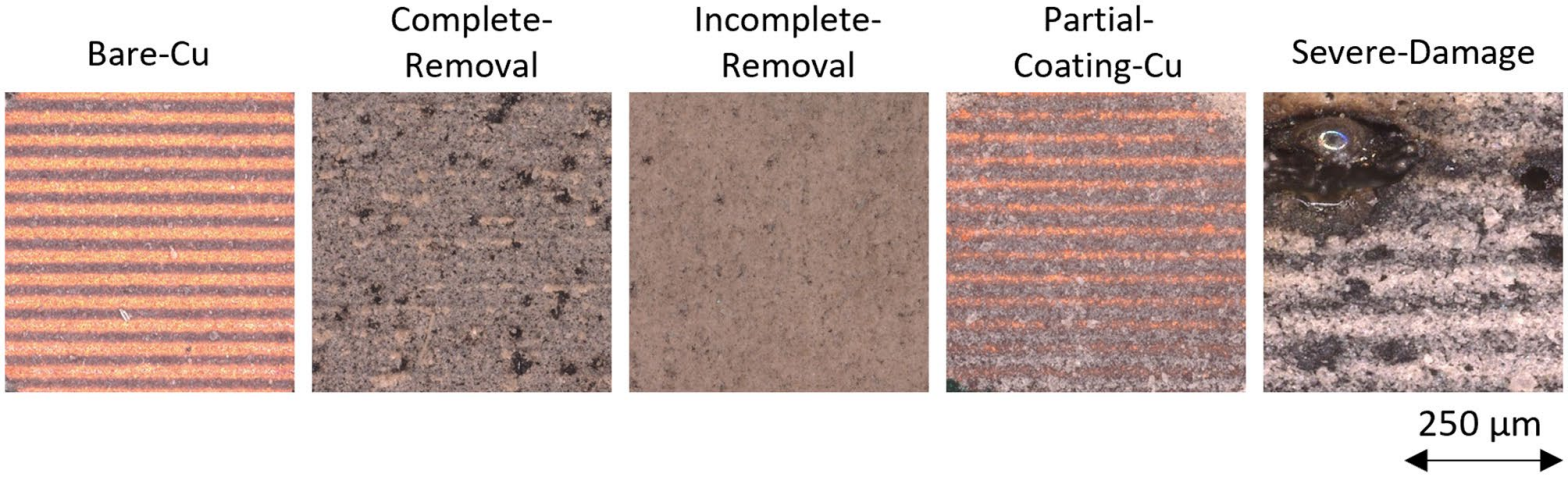
Microscopy images of the different annotation classes: Bare-Cu, Complete-Removal, Incomplete-Removal, Partial-Coating-Cu, Severe-Damage.

Quality annotation plays a pivotal role in the effectiveness and reliability of DL models. Several factors have made labeling challenging during the annotation process in this study. Initially, the uneven cleaning of the coatings results in smooth boundaries between categories, making it difficult for annotators to delineate the contours of each category. Furthermore, the categories Partial-Coating-Cu and Bare-Cu are hard to distinguish due to their similar colors and lack of distinct features. Additionally, assessing the extent of substrate overtreatment is challenging, meaning the distinction between severe damage and complete removal is difficult to quantify. Based on microscopic observations, a detailed and scientific annotation guideline was established to address these issues (see Fig. 2).

The annotation tool employed is Roboflow^27^. Roboflow is a versatile image management tool that offers an intuitive interface, support for multiple annotation formats, automation of repetitive tasks, and seamless team collaboration.

#### Dataset preparation and augmentation

**Figure 3:**
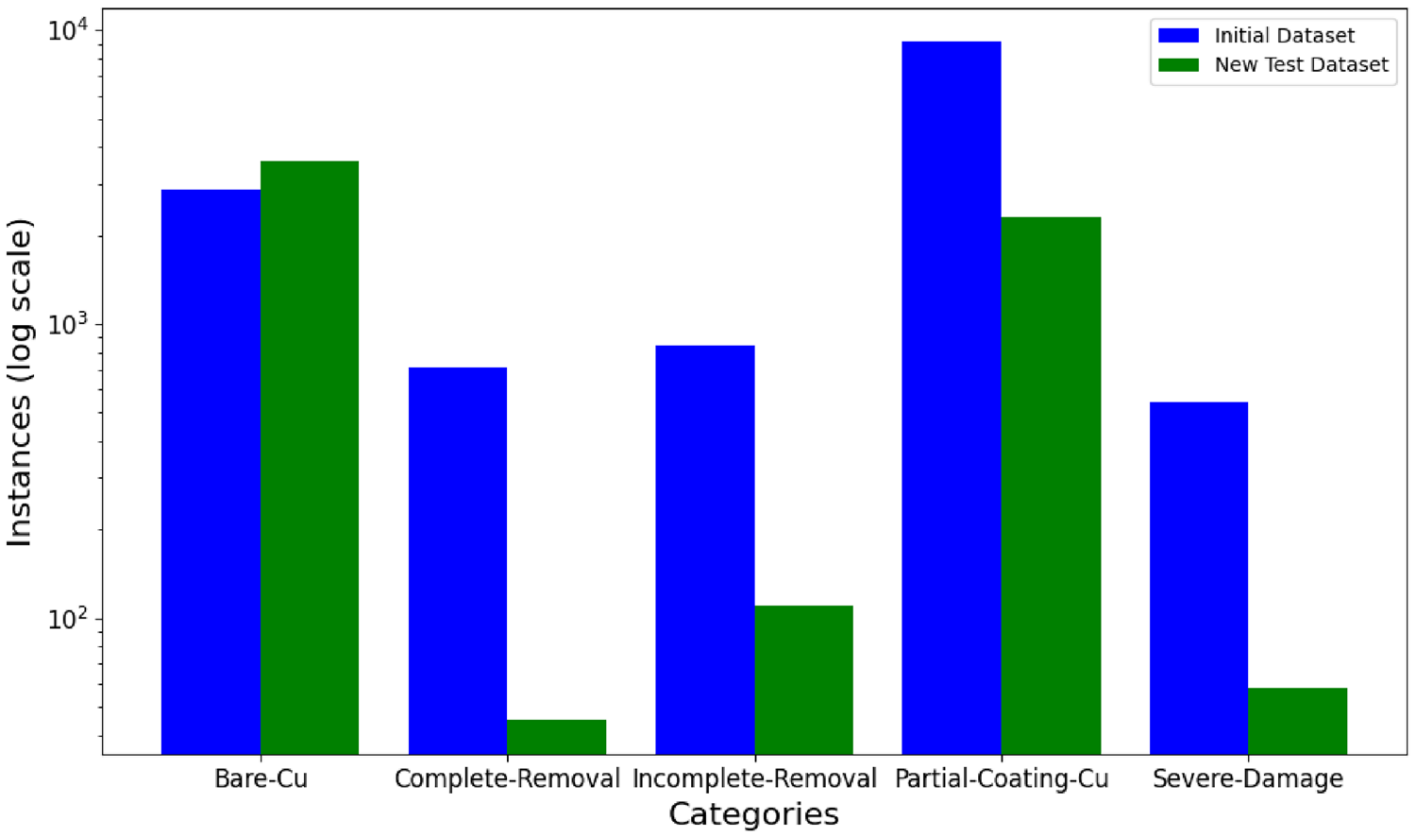
Comparison of category distributions in initial and new test datasets for PCB segmentation tasks (log scale).

The initial dataset comprised 700 images, each with a resolution of 640$\times$640 pixels. In our labeled dataset, the number of instances is not balanced. Figure 3 illustrates the distribution of instances for the five categories in the initial dataset and the new test dataset, and a comparison plot on a logarithmic scale highlights the significant differences. If improperly handled, this bias could lead to model overfitting towards the largest class^28^.

The dataset was partitioned into training (88%), validation (8%), and test (4%) sets. This distribution maximized the training data while ensuring sufficient samples for validation and independent testing to evaluate the model’s performance accurately. Image data augmentation^29^ is employed uniformly across all classes to artificially expand the scale of the train dataset^30^. Although this approach does not directly alter the class distribution or fully mitigate class imbalance, artificial dataset expansion can still help reduce overfitting^31^, improve model performance, and enhance the model’s generalization ability^32^. The applied augmentations included horizontal and vertical flips to address variations in defect orientation, 90° rotations (clockwise and counter-clockwise) to simulate different rotational alignments, and brightness adjustments ranging from -15% to +15% to mimic changes in lighting conditions. The same training, validation, and test datasets will be employed concurrently to train and evaluate the performance of both YOLOv8-seg and Mask R-CNN algorithms.

### Training device and parameters

Dataset training, validation, and testing were conducted using Google Colaboratory (Google Colab). This free Jupyter Notebook environment eliminates software installation and provides access to high-performance GPUs for intensive computational tasks. For this study, the NVIDIA A100 GPU available through Colab was employed to handle the computational demands.

We implemented the Mask R-CNN model using Detectron2, a platform developed by Facebook AI Research noted for its flexibility, speed, and modular architecture^33^. Additionally, the YOLOv8-seg model and its weights were sourced from GitHub^34^, enhancing the transparency and reproducibility of our work.

This study leveraged Mask R-CNN and YOLOv8-seg across differing configurations and scales. Mask R-CNN was tested utilizing ResNet-50 and ResNet-101 as backbones, while YOLOv8-seg was analyzed on ‘small’ (s) and ‘large’ (l) scales. This choice of configurations was carefully made to balance computational efficiency and model performance. For Mask R-CNN, ResNet-50 and ResNet-101 were selected as backbones due to their ability to achieve a desirable trade-off between accuracy and resource usage. Smaller backbones, such as ResNet-18 and ResNet-34, were excluded as they are better suited for simpler tasks or smaller datasets. Conversely, larger backbones like ResNet-152 were avoided due to their substantial computational requirements, which are impractical for real-time applications. Similarly, for YOLOv8-seg, the “small” (s) and “large” (l) configurations were chosen to represent two ends of the efficiency-accuracy spectrum, where “s” excels in lightweight, real-time performance, and “l” offers enhanced accuracy and generalization with more complex architecture. These selections ensured an optimal balance tailored to the specific requirements of this study. To ensure a consistent and unbiased comparison, all models were trained for up to 320 epochs under uniform conditions, using a batch size of 8. An early stopping mechanism was implemented for YOLOv8-seg, halting training if no improvement was observed over 100 epochs. For the optimizer, we adhered to model-specific recommended settings: Mask R-CNN employed the Stochastic Gradient Descent (SGD) optimizer, while YOLOv8-seg utilized an ‘auto’ setting that dynamically selects the optimal optimizer based on training dynamics, typically starting with AdamW for early rapid convergence and switching to SGD for improved fine-tuning and generalization as training advanced^35^. Our adherence to official guidelines ensures a fair comparison, confirming that performance differences are due to the inherent properties of the model architectures, not external training variations.

### Metrics

Monitoring the loss during the training and validation processes is crucial. It aids in determining model convergence and provides immediate feedback on the model’s performance on both the training and validation datasets, enabling the detection of overfitting or underfitting. This study delves into the dynamic changes in loss parameters within the YOLOv8-seg and Mask R-CNN frameworks throughout the training and validation process.

For YOLOv8-seg, there are four primary loss parameters: bounding box loss (Box_Loss), classification loss (Cls_Loss), segmentation loss (Seg_Loss), and distribution focal loss (DFL_Loss)^36,37^. Box_Loss measures the accuracy of bounding box predictions regarding their coordinates and dimensions relative to the actual objects. Cls_Loss quantifies the discrepancy between the model’s predicted probabilities for object classes and the actual class labels. Seg_Loss examines the alignment between the model’s predicted segmentation masks and the ground truth masks, evaluating the model’s performance in the semantic segmentation task. DFL_Loss addresses class imbalance by placing greater importance on less frequently occurring classes^38^. In the Mask R-CNN framework, the total loss (Total_Loss) comprises several components, each targeting a specific aspect of the model’s predictions. The classification loss (Cls_Loss) and bounding box loss (Box_Loss) are similar to those in YOLOv8-seg. The mask loss (Mask_Loss) involves calculating the cross-entropy loss between the predicted mask and the true mask for each instance, which is essential for pixel-level segmentation^21,38,39^. Additionally, the model incorporates RPN classification loss and RPN box loss, which is crucial for training the Region Proposal Network (RPN). These losses ensure that the RPN generates high-quality region proposals consistent with the actual labels, enhancing the model’s object detection and segmentation capabilities^40^.

Several key metrics are commonly used to evaluate the performance of target detection algorithms. Precision measures the model’s ability to correctly identify positive instances during prediction, while recall measures the proportion of actual positives correctly identified by the model^41^. Average Precision (AP) is the area under the precision-recall curve, with recall on the x-axis and precision on the y-axis. Mean Average Precision (mAP)^42^ measures the average precision across all categories and multiple Intersections over Union (IoU) thresholds. IoU^43^ indicates the ratio of the intersection area between the ground truth and prediction to the area of their union^44^. This study typically employs mAP50 (box), mAP50(seg)^45^, and frames per second (FPS) to assess algorithm performance in target detection. mAP50 (box) represents the average precision of all classes when IoU is 0.5 for bounding box predictions, while mAP50 (seg) represents the average precision for segmentation tasks at the same IoU threshold. Processing speed is determined by the reciprocal of the time required to process one frame.

## Results and evaluation

### Models’ performance

**Figure 4:**
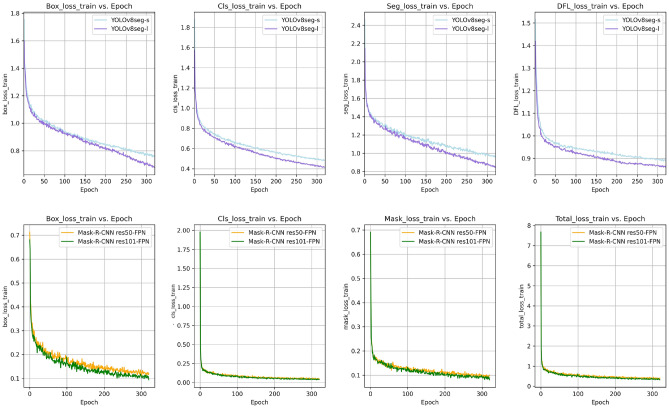
Loss curves for the training dataset.

Figure 4 illustrates the training loss across epochs for all models, indicating a general downward trend, signalling that the models effectively learned from the training dataset. Notably, the Mask R-CNN model manifested a lower loss for all parameters, suggesting superior performance on the training set. This superiority can be attributed to the architecture of Mask R-CNN, specifically designed for precise object detection and instance segmentation, which might be more adept at handling complex scenes and detailed spatial tasks. However, while the lower loss suggests that Mask R-CNN might perform better on similar unseen data, further evaluation using validation and test datasets is necessary to determine if this translates into better generalization capabilities than YOLOv8-seg while also considering potential overfitting issues.

**Figure 5:**
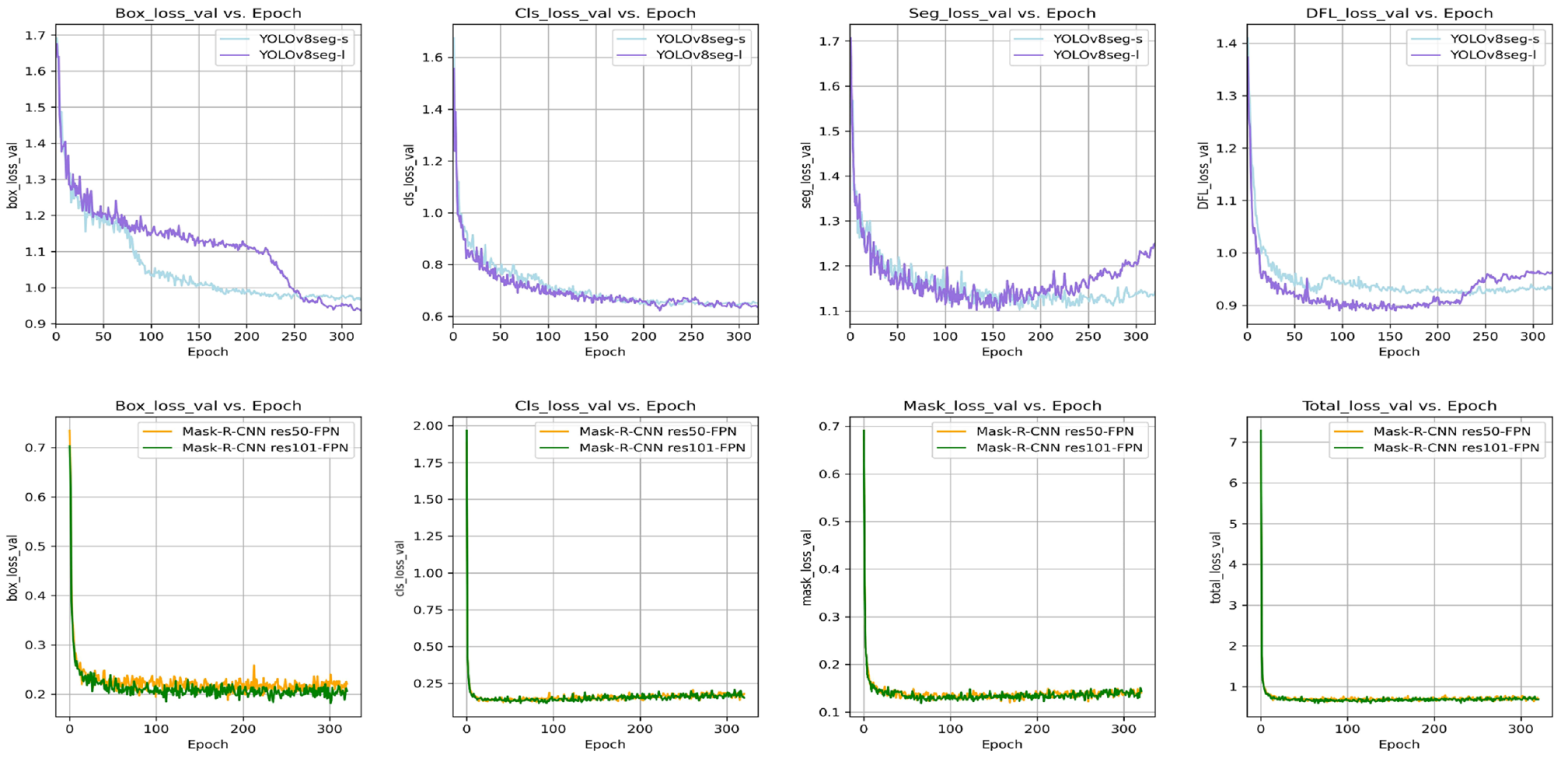
Loss curves for the validation dataset.

Figure 5 shows the validation loss curves, critical for evaluating the models’ ability to generalize to new data. The Mask R-CNN models’ validation loss is closely aligned with their training loss, which indicates well-balanced, effective model training. Conversely, YOLOv8-seg presented a more substantial discrepancy between training and validation loss, especially regarding Seg_loss and DFL_loss. This could imply that YOLOv8-seg might have been overfitting to the training data or that the training set lacked the diversity required for the model to generalize effectively. Mask_Loss and the Total_Loss were only applicable to the Mask R-CNN model. We observed that after an initial rapid decrease, these curves plateaued on both the training and validation datasets, indicating no significant progress after a certain point in learning the segmentation mask. Data limitations are likely contributing to the plateau. To enhance the generalization capabilities of our model, we will investigate advanced regularization techniques, enlarge the training dataset, and implement sophisticated data augmentation strategies.

To evaluate the effectiveness of the models, we utilized several performance metrics as detailed in Table 1. These metrics include mAP50 (seg) and mAP50 (box), the parameter count and processing speed, measured in frames per second (FPS). Metrics mAP50 (seg) and mAP50 (box) are reported in percentage (%) and are based on the models’ performance on the original test dataset. The number of parameters directly impacts computational efficiency and real-time suitability. Models with fewer parameters, like YOLOv8-seg(s), provide faster inference and require fewer computational resources, making them well-suited for real-time applications. While the parameter counts for YOLOv8-seg(l) (46.0M) and Mask R-CNN with a ResNet-50 backbone (44.0M) are similar, their differences in performance underscore the critical role of architectural design. YOLOv8-seg(l)’s streamlined architecture allows it to achieve faster processing speeds without a significant loss in segmentation accuracy, whereas Mask R-CNN leverages its design to deliver slightly higher precision at the cost of slower inference. This comparison highlights how both parameter count and architectural efficiency together shape the balance between accuracy and computational efficiency. The Mask R-CNN model, incorporating a ResNet-50 backbone, achieved an mAP50 (seg) of 84.097%, demonstrating the highest precision in segmenting PCB surface areas. However, it exhibited a slower processing speed at 1.52 FPS, which limits its scalability for high-speed applications. In contrast, YOLOv8-seg(l) leveraged its optimized architecture to achieve a balance between precision and speed, making it a more practical choice for applications that demand both real-time responsiveness and high segmentation performance. The YOLOv8-seg, configured on a ‘small’ scale, achieved the fastest processing speed at 7.12 FPS, with 11.8 million parameters. It maintained mAP50 (seg) and mAP50 (box) scores of 81.3% and 85.0%, respectively, meeting the segmentation precision requirements of this study. Notably, YOLOv8-seg slightly outperformed the Mask R-CNN in detection precision, achieving an mAP50 (box) of 85.5%, indicating its viability for applications that demand superior detection precision. Future work will explore more advanced algorithms or further optimization of existing models to enhance segmentation precision, increase processing speeds, or achieve a better balance between the two.

**Table 1:** PCB segmentation model performance metrics evaluation.

**Segmentation algorithms**	**Backbone or scale (Parameters in M)**	**mAP50 (seg) in %**	**mAP50 (box) in %**	**Processing speed in FPS**
Mask R-CNN	ResNet-50 (44.0M)	84.097	84.567	1.52
	ResNet-101(63.0M)	83.685	82.824	1.53
Yolov8-seg	s (11.8M)	81.3	85.0	7.12
	l (46.0M)	82.8	85.5	3.98

### Visualization and analysis

This section presents a comparative analysis of visualization results from two segmentation models, YOLOv8-seg (l) and Mask R-CNN (ResNet-50), utilizing two sets of representative images from the test dataset. The visualization of the test results shows only recognized objects with a confidence level of 0.5. Each column in the representative image represents the original image, the annotated image, the segmentation result by YOLOv8-seg (l), and the segmentation result by Mask R-CNN (ResNet-50).

**Figure 6:**
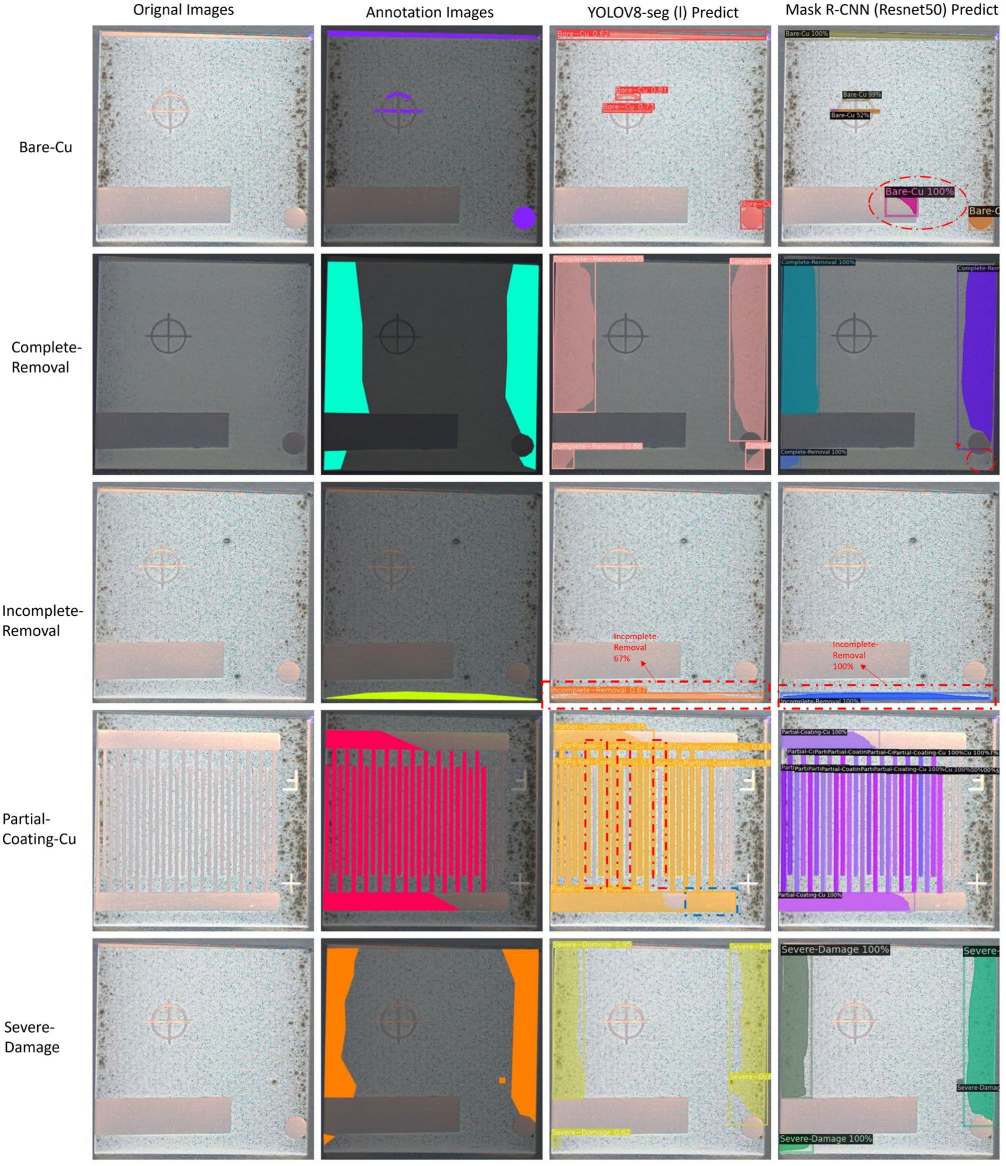
The segmentation outcomes for various categories under YOLOv8-seg and Mask R-CNN. Each row represents a unique PCB condition, including Bare-Copper (Bare-Cu), Complete-Removal, Incomplete-Removal, Partial-Coating of Copper (Partial-Coating-Cu), and Severe-Damage. Each column represents the original image, the annotated image, the segmentation result by YOLOv8-seg, and the Mask R-CNN (same below). Images were captured after each cleaning cycle, with a total of 25 cycles conducted to ensure the presence of as many classes as possible. In annotation images, purple areas represent Bare-Cu, blue areas signify Complete-Removal, yellow areas are indicative of Incomplete-Removal, pink areas denote Partial-Coating-Cu, and orange areas correspond to Severe-Damage (same below).

The first set (Fig. 6) compares the models’ segmentation results on the PCB surface. As can be seen in the first row of Fig. 6, both the Mask R-CNN model and the YOLOv8 model correctly classify and segment the bare copper regions. The output of the YOLOv8-seg model exactly matches the annotations. In contrast, the Mask R-CNN over-predicts one bare copper instance (red circle in the first row), which is not included in the annotations, suggesting a tendency for the Mask R-CNN to over-segment the bare copper regions and a risk of misclassification that may affect the processing of the copper regions. For “complete removals”, both models are correctly detected and labeled. The Mask R-CNN has a slightly higher confidence than the YOLOv8-seg. The mask provided by the Mask R-CNN lacks a “completely removed” region (red circle in the second row), which may be because the texture and features of this region are not distinct enough compared to the other completely removed regions, revealing the challenges of such detection. Both trained models segment “incomplete removal” correctly, with the Mask R-CNN showing higher confidence (100%) in the “incomplete removal” prediction compared to the cautious 67% confidence of YOLOv8-seg. Mask R-CNN correctly segmented “Partial-Coating-Cu” with high confidence, but the mask provided by YOLOv8-seg is missing three small parts of the “Partial-Coating-Cu” region (red rectangles in the fourth row), which have fuzzy edges compared to the other parts that are correctly segmented. In addition, YOLOv8-seg incorrectly predicted and segmented an area in the lower portion of the image (the blue square in the fourth row) as “Partial-Coating-Cu”. This may be due to the oxidation of copper, which causes this instance to be significantly less bright than other instances of the same type. Both models successfully segment the “Severe Damage” regions, highlighting their training advantages. In this image, the “severely damaged” regions are easily distinguished from the “completely removed” regions.

**Figure 7:**
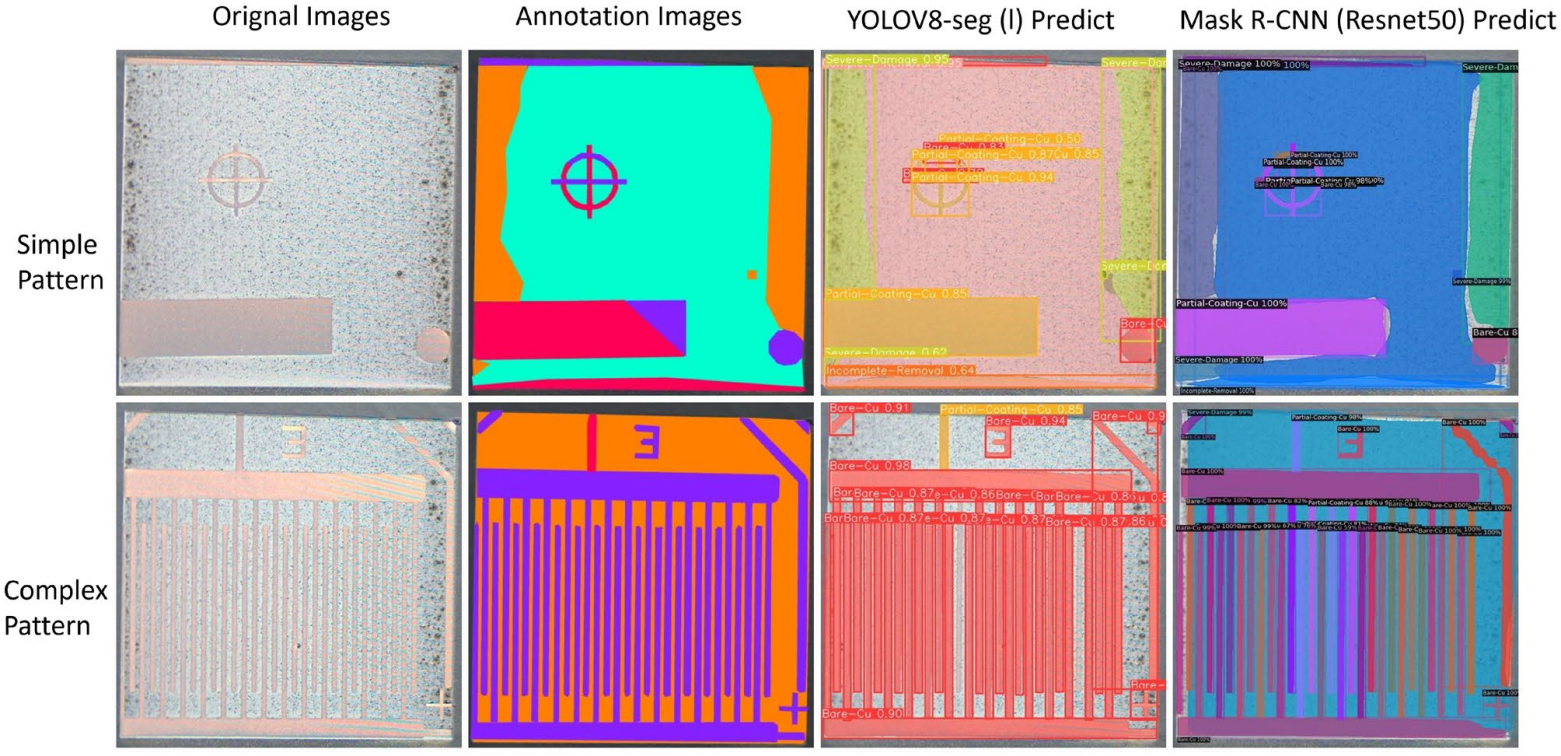
The segmentation results on PCB surfaces with simple and complex patterns.

The second set of results (see Fig. 7) compares segmentation performance on PCB surfaces with simple and complex patterns. Regarding the simple pattern, the segmentation results from Mask R-CNN and YOLOv8-seg align very closely with the actual object shapes. For the complex pattern, YOLOv8-seg appears to miss some object areas that Mask R-CNN captures, such as the ‘Severe-Damage’ area (marked orange in the annotation image), potentially indicating a lower recall for YOLOv8-seg. Conversely, Mask R-CNN segments more details, suggesting it may be advantageous in handling complex pattern segmentation tasks. For the post-cleaning surface quality assessment, the analysis of segmentation results ensures the thoroughness of the cleaning process. Mask R-CNN’s ability to recognize nuanced conditions and its higher detection rate in complex patterns suggest its more suitability for detailed assessments of post-cleaning quality.

In summary, the visualisation performance of YOLOv8-seg and Mask R-CNN on the initial test set shows that they can successfully fulfil the requirements of real-time segmentation guidance and post-cleaning quality assessment in PCB laser cleaning applications. YOLOv8-seg excels in precision, minimizing false positives and accurately identifying central Bare-Cu areas without over-segmentation, ensuring precise localization. In contrast, Mask R-CNN demonstrates higher recall, capturing more relevant objects and segmenting more details, particularly in complex patterns. However, Mask R-CNN tends to over-segment areas like Bare-Cu, leading to potential misclassifications. For laser cleaning monitoring, YOLOv8-seg is more suitable due to its high precision and effective avoidance of false positives, making it better suited to the precise and reliable requirements of the process. To enhance the scientific validity of this study, in the next section, the two models will be applied to a new dataset with a brand new pattern to verify their feasibility on laser cleaning tasks.

### Models’ performance on new test dataset

**Figure 8:**
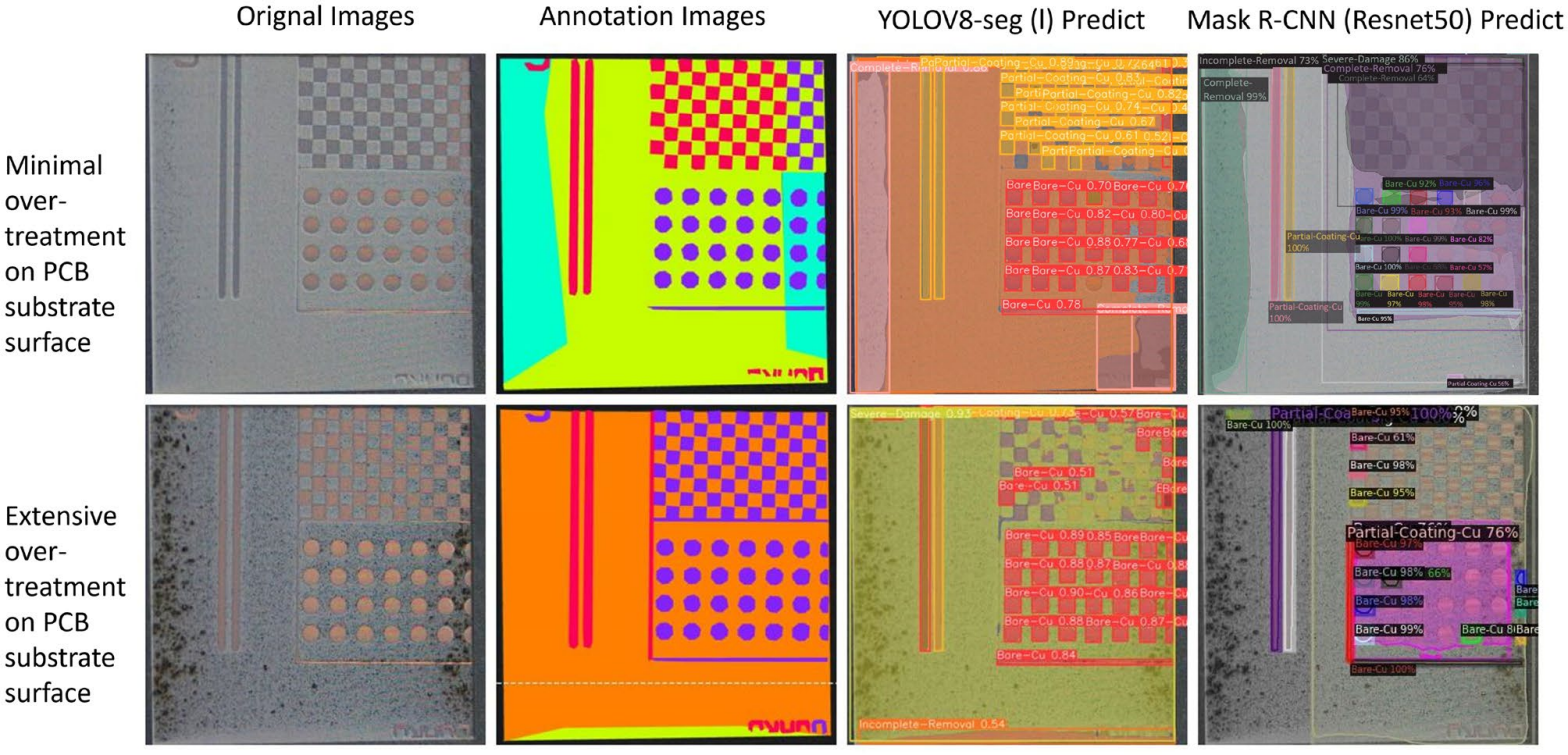
Model visual performance on the new test dataset.

This study employed a new test dataset with distinct PCB patterns and surface impurities, diverging significantly from our training and initial test sets (Fig. 8). This testing approach reinforces our model’s generalization assessment and rigorously evaluates its robustness across different contexts.

On the new test dataset, the YOLOv8-seg model achieved an mAP(seg) of 69%, significantly surpassing the Mask R-CNN model, which registered an mAP(seg) of 36.91%. The performance of YOLOv8-seg was particularly notable in its precise identification of diverse categories, such as the ‘checkerboard’ pattern, not initially included in the training dataset, as depicted in Fig. 8. Conversely, Mask R-CNN exhibited pronounced difficulties in recognizing such new patterns, highlighting its inadequacies in generalizing beyond familiar training features. Initial speculation suggests that this difference in performance is mainly due to the different architectural approaches adopted by the two models. Mask R-CNN, a region-based convolutional network, depends on localized feature detection and accurate bounding box localization, making it suitable for familiar datasets but less adaptable to new and diverse inputs. In contrast, YOLOv8-seg adopts a holistic strategy, processing the entire image via an end-to-end single-stage detection architecture. This design facilitates the integration of global contextual information, substantially improving its ability to generalize across varied and dynamic environments.

**Figure 9:**
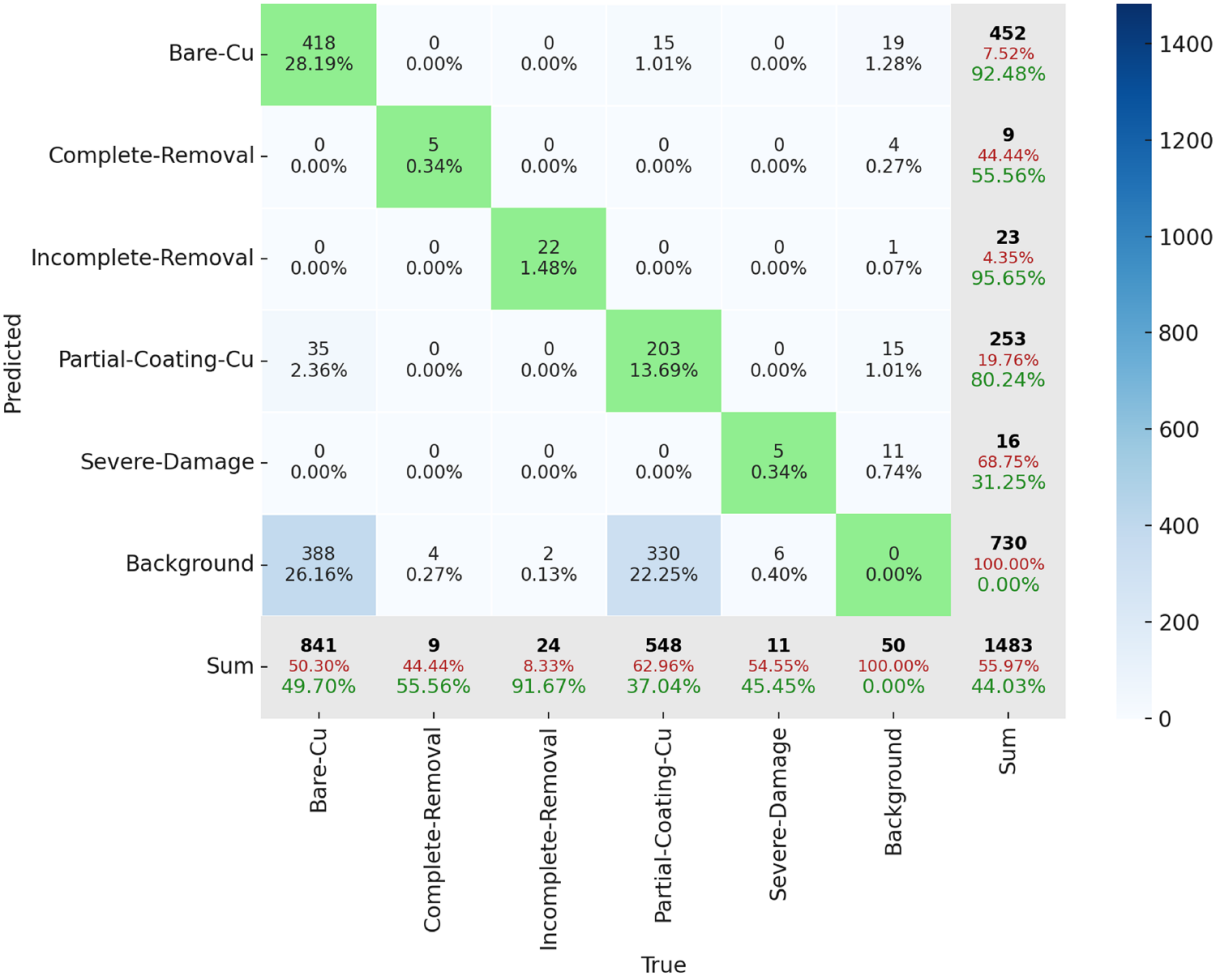
Confusion matrix of YOLOv8-seg. The x-axis represents the ground truth classes, and the y-axis represents the predicted classes. Green-colored cells indicate correctly classified instances (true positives) and are highlighted for emphasis, while other cells are shaded using a blue gradient scale according to the legend. Row sums (rightmost column) show the total number of predictions for each class. Each includes a green percentage indicating the proportion of correct predictions and a red percentage for incorrect predictions. Column sums (bottom row) show the total number of actual instances per class, with green percentages indicating the proportion correctly identified and red percentages for misclassified instances. Each individual cell also displays a percentage value, representing the proportion of the total test dataset (1483 instances) that the specific prediction accounts for.

Due to the observed limitations of Mask R-CNN in adapting to new test datasets, the YOLOv8-seg model was selected for critical tasks in our study, real-time segmentation for laser cleaning guidance and post-cleaning surface quality assessment. Although YOLOv8-seg demonstrated significantly better segmentation performance overall, it is essential to analyze its classification accuracy across different categories. To gain deeper insights, we examined its confusion matrix (Fig. 9) to identify class distribution and interclass confusion. The analysis of the confusion matrix indicates that among 24 instances of ‘Incomplete-Removal’, the model correctly detected 22 instances, while 2 instances were misclassified as ‘Background’. For ‘Incomplete-Removal’, the row and column sums are 23 and 24, respectively. Regarding the sum row, the 22 correctly predicted instances account for 95.65% of the 23 total predictions made for ‘Incomplete-Removal’, leaving an error of 4.35%. On the other hand, in the sum column, 91.67% of the 24 ground truth ‘Incomplete-Removal’ were correctly classified, while 8.33% were misclassified. The bottom-right value of the matrix represents the total number of instances, which is 1483. The sum of true positive classifications across all categories is 653, accounting for 44.03% of the total predictions. Given the complex patterns in each class, the system tends to exhibit confusion when distinguishing between different categories. The complexity of background conditions appears to be a major factor contributing to errors, leading to increased false positives and false negatives in most cases. Additionally, the model frequently confuses Partial-Coating-Cu with Bare-Cu, as indicated by 35 misclassifications, likely due to their similar visual characteristics. In future research, efforts should be directed toward expanding the diversity of background data, refining feature extraction techniques to enhance class differentiation, and implementing class-balanced learning strategies. These improvements may help reduce misclassification, minimize confusion between visually similar categories, and improve overall detection performance.

Table 2 presents the mAP50 (seg) scores for individual categories across the initial and new test datasets. This detailed breakdown allows for a class-specific evaluation of the model’s segmentation performance, highlighting variations in precision among different categories. For laser cleaning guidance, YOLOv8-seg consistently segments ‘Incomplete-Removal’ and ‘Partial-Coating-Cu’ categories across various test sets, as documented in Table 2. This segmentation capability is most effective when substrates are minimally damaged (third column of the first row of Fig. 8), which aligns with laser cleaning guidance requirements. However, when over-processing the substrate results in the blurring of features such as texture, colour, and boundaries, and the segmentation precision of the model is significantly degraded (third column of the second row of Fig. 8). Since over-processing can and should be avoided in practice, this limitation does not significantly affect the overall usability of the model. For the task post-cleaning surface quality assessment, the model’s segmentation precision for the “Severe-Damage” category becomes particularly important. However, the performance of the YOLOv8-seg model in this category is lower in the new test sets compared to the initial test dataset. This limitation is mainly due to the training dataset containing relatively few samples of the “Severe-Damage” category, which affects the model’s ability to generalize effectively, resulting in reduced segmentation precision for this category. Despite this limitation, in practical production processes, severe damage to substrates can be largely avoided with appropriate cleaning settings and the previously mentioned laser cleaning guidance task, reducing the impact of this issue on the model’s performance in executing the post-cleaning surface quality assessment task. Future research will enhance YOLOv8-seg’s robustness and generalization by diversifying the training dataset and refining parameters and structural design to improve performance across various conditions.

**Table 2:** Comparison of mAP50(seg) scores in % for YOLOv8-seg model across different test datasets.

Class	Initial test dataset	New test dataset
Bare-Cu	68.4	77.3
Complete-Removal	85.5	60.3
Incomplete-Removal	96.7	98.3
Partial-Coating-Cu	86.6	58.3
Severe-Damage	76.7	50.6

## Discussion

Recent advancements in PCB defect detection have demonstrated the potential of DL models in handling a variety of surface conditions. For example, YOLO-MBBi^46^ and Faster R-CNN-based approaches^47^ have shown success in detecting surface defects with high precision. Similarly, lightweight models such as YOLO-pdd^48^ have been proposed to address real-time requirements for PCB defect detection, while Zhang et al.^49^ explored X-ray imaging for solder joint defect detection, highlighting the adaptability of DL techniques to different imaging modalities. These successful applications provide a foundation for addressing the unique challenges of our study, which targets more complex PCB surfaces characterized by coatings, oxidized copper lines, and variable surface textures. The variability and occlusion introduced by such conditions require models capable of both precise segmentation and robust generalization, motivating our exploration of YOLOv8-seg and Mask R-CNN for this application. The capabilities and limitations of two advanced DL algorithms, YOLOv8-seg and Mask R-CNN, are evaluated for PCB surface segmentation in laser cleaning monitoring. The segmentation results for the two algorithms on the initial dataset were satisfying. YOLOv8-seg demonstrates superior performance, evidenced by a higher frame per second (FPS) rate, making it particularly suitable for industrial applications where time efficiency is critical. Mask R-CNN offers a more nuanced interpretation through its two-stage process, capturing the complexity of PCB surfaces with greater precision. However, due to its architectural design, Mask R-CNN underperforms on the new test set compared to YOLOv8-seg when handling new patterns. YOLOv8-seg is selected for future projects based on its robust generalization capabilities, high precision, and ability to meet the real-time demands of laser cleaning processes. It can dynamically adjust laser cleaning strategies, maintaining the precision required to treat specific instances of residue while safeguarding sensitive PCB areas. In addition, in analyzing the visualization results of the initial test set, we found that the two algorithms have different visualization segmentation results for each category. Our study also has several limitations. Firstly, the segmentation models are trained on a limited set of labeled categories, restricting their ability to identify and segment diverse PCB surface state features accurately. Secondly, the primarily light-rich environment of the current dataset may impair model performance under the low or fluctuating lighting conditions typical in industrial settings. Additionally, while the models show some capability in recognizing PCB surface states, their robustness against complex and challenging scenarios needs further validation.

### Further work

In order to address the previously identified deficiencies in the model, further research will investigate the reasons for differing segmentation results. This investigation will focus on factors such as model architectures, data preprocessing and augmentation, loss functions, and handling of background interference and noise. It will explore the internal mechanisms of the models by examining intermediate feature maps and layer activations. In the context of industrial environments, where low or fluctuating light conditions are the norm, it would be advantageous to investigate the impact on models incorporating datasets encompassing a range of lighting scenarios. This would ensure improved model performance in less than ideal conditions. Additionally, Future research should employ larger datasets to improve model performance. This includes incorporating additional defect types, such as residues and spots/oxides, and enriching the dataset with diverse surface patterns and textures. While the models show some capability in recognizing PCB surface states, their robustness against complex and challenging scenarios needs further validation. Addressing scenarios involving diverse contaminants or subtle patterns, which can significantly challenge accurate segmentation, may require integrating models with advanced feature detection and segmentation capabilities. Planned improvements include enhancing feature extraction capabilities through attention mechanisms (e.g., CBAM) and multi-scale feature fusion, optimizing the loss function with IoU-based metrics for better segmentation accuracy, and addressing class imbalance using techniques such as Focal Loss and dynamic sampling. Real-time performance will also be improved through pruning and quantization to ensure suitability for industrial applications. Finally, Explainable Artificial Intelligence (XAI) techniques, such as Grad-CAM and SHAP, will be incorporated to enhance model interpretability and provide actionable insights into decision-making processes.

Developing an innovative automated laser cleaning monitoring system for decoating PCBs is central to our future initiatives. This proposed system integrates a laser unit with a camera controlled by TwinCAT automation software, enhancing its ability to clean and simultaneously monitor the PCB surfaces. Following a cleaning cycle, the camera captures high-definition images processed by an advanced DL model. Embedded within the TwinCAT environment, this pre-trained DL model performs critical tasks such as detecting areas requiring re-cleaning and advising on adjustments to the laser unit’s parameters. This dynamic adaptation optimizes the cleaning cycles, ensuring thorough cleaning of PCB coatings without damaging the substrate. The findings on precision and speed described in this paper are used to achieve the highest possible accuracy within the available computing time of 700 ms. Experiments have shown that an accuracy of over 80 % is sufficient for initial laboratory tests. This is due to the fact that the paint stripping of the PCB is less sensitive, and overtreatment only occurs after the laser processing cycle has been repeated around three times. The algorithm can, therefore, check the areas on the board up to three times before damage occurs. It has been demonstrated that the precision of Yolov8-seg is already adequate for this purpose. Nevertheless, it is planned to increase the accuracy to over 90 % in the future in order to avoid unnecessary treatment cycles - especially in industrial applications - and thus further reduce the processing time.

**Figure 10:**
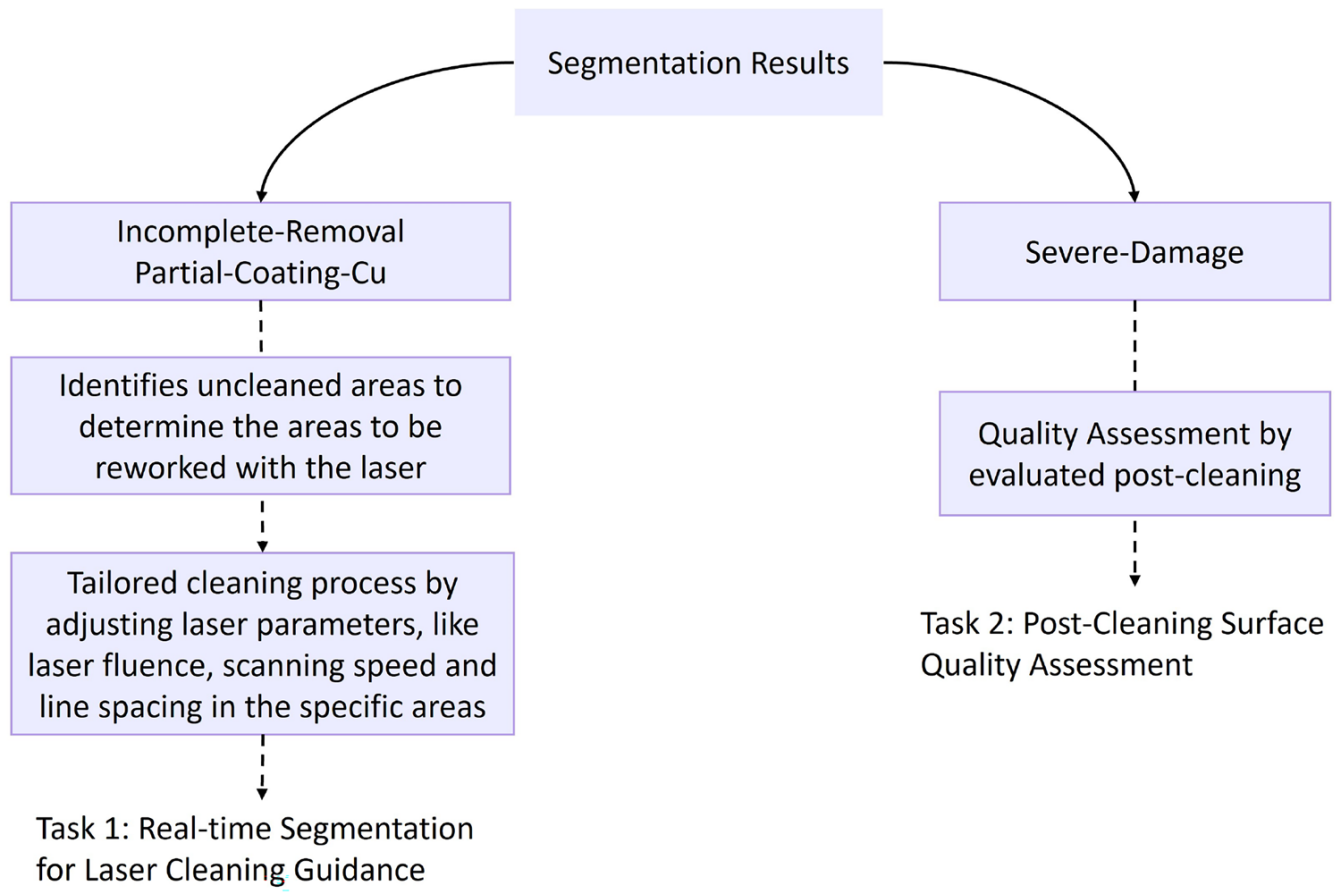
Flowchart of real-time segmentation and post-cleaning assessment in laser cleaning processes. (Implemented steps are shown with solid lines, while future work is indicated with dashed lines.).

The presented study will be dedicated to advancing two engineering tasks in laser cleaning processes through segmentation results (Fig. 10): the first task will be real-time segmentation for laser cleaning guidance. In this task, the camera will capture high-resolution images of the PCB surfaces in real-time during laser cleaning. Through the segmentation results for ‘Incomplete-Removal’ and ‘Partial-Coating-Cu’, the laser scanner will dynamically adapt its cleaning strategies in real-time. The areas identified as requiring further cleaning will be targeted and cleaned again. This will help avoid the need for the entire surface to be reprocessed, thus preventing over-treatment. Furthermore, the processing time will be significantly reduced in this way. This will also ensure that each specific instance of coating residue is treated appropriately. At the same time, areas classified as ‘Complete-Removal’, ‘Severe-Damage’, and ‘Bare-Cu’ will be recognized and bypassed, preserving the bare copper and PCB substrate’s integrity. The second task, post-cleaning surface quality assessment, is expected to focus on assessing the quality of the cleaned surfaces after the final treatment by evaluating the ‘Severe Damage’ areas. This will enable the creation of a final quality control measure for the treated component and thus enable 100 % quality assurance.

## Conclusions

This study initiates developing an artificial intelligence-based system for monitoring laser-cleaned PCBs, employing an image segmentation algorithm that enables real-time analysis of PCB surface states. By precisely identifying areas that require cleaning, segmentation facilitates targeted laser processing, minimizing unnecessary operations and significantly reducing processing time. Additionally, accurate segmentation supports post-cleaning surface quality assessment by providing detailed insights into the effectiveness of the cleaning process and identifying any residual defects. This functionality is crucial for real-time segmentation for laser cleaning guidance and post-cleaning surface quality assessment, significantly advancing intelligent manufacturing systems by enhancing PCB recycling processes’ efficiency, quality, and sustainability. For the development of an artificial intelligence-based system for monitoring laser-cleaned PCBs in real-time, we trained and evaluated four DL models-YOLOv8-seg (s), YOLOv8-seg (l), Mask R-CNN (ResNet50), and Mask R-CNN (ResNet101). These models were assessed on their ability to identify and segment different states on PCB surfaces post-cleaning, using metrics such as mAP50 (seg), mAP50 (box), and FPS. Results showed that Mask R-CNN with a ResNet-50 backbone achieved an mAP50 (seg) of 84.097% at 1.52 FPS, while YOLOv8-seg (l) recorded an mAP50 (seg) of 82.8% at 3.98 FPS. To enhance the science, the trained models were then tested on the new test dataset. The results highlight both the strengths and limitations of the proposed models. YOLOv8-seg (l) demonstrated strong generalization across all categories, achieving an overall mAP50 (seg) of 69%. However, performance on rare defect types, such as Severe-Damage, was lower at 50.6%, indicating the need for further optimization, such as targeted augmentation or advanced loss functions to address the class imbalance and enhance robustness. In contrast, Mask R-CNN (ResNet-50) achieved a significantly lower overall mAP50 (seg) of 36.91%, making it less suitable for further applications. YOLOv8-seg was selected for future projects due to its superior generalization and segmentation performance. These findings underscore the potential of these models for real-time segmentation and surface quality assessment in laser cleaning applications. Additionally, this study has provided a publicly accessible, labeled dataset, supporting further research. The trained models have proven effective in enhancing the precision of laser cleaning processes, can be contributing to more sustainable recycling practices for Waste Electrical and Electronic Equipment (WEEE). This work demonstrates the effectiveness of DL models in a novel application area and lays the groundwork for future research aimed at refining these models and integrating them into more complex and adaptive laser cleaning systems. This advancement is a crucial step toward the development of automated recycling and repair concepts for components on PCBs, ultimately helping to reduce resource consumption and minimize electronic waste generation.

## Data Availability

The data that support the findings of this study are available from the corresponding author, M.V., upon reasonable request.
